# Recent Progress on Electrical and Optical Manipulations of Perovskite Photodetectors

**DOI:** 10.1002/advs.202100569

**Published:** 2021-05-24

**Authors:** Fang Wang, Xuming Zou, Mengjian Xu, Hao Wang, Hailu Wang, Huijun Guo, Jiaxiang Guo, Peng Wang, Meng Peng, Zhen Wang, Yang Wang, Jinshui Miao, Fansheng Chen, Jianlu Wang, Xiaoshuang Chen, Anlian Pan, Chongxin Shan, Lei Liao, Weida Hu

**Affiliations:** ^1^ State Key Laboratory of Infrared Physics Key Laboratory of Intelligent Infrared Perception Shanghai Institute of Technical Physics Chinese Academy of Sciences Shanghai 200083 China; ^2^ University of Chinese Academy of Sciences Chinese Academy of Sciences Beijing 100049 China; ^3^ Key Laboratory for Micro‐Nano Optoelectronic Devices of Ministry of Education and Hunan Provincial Key Laboratory of Low‐Dimensional Structural Physics and Devices School of Physics and Electronics Hunan University Changsha 410082 China; ^4^ Terahertz Technology Innovation Research Institute Terahertz Spectrum and Imaging Technology Cooperative Innovation Center Shanghai Key Lab of Modern Optical System University of Shanghai for Science and Technology Shanghai 200093 China; ^5^ Hangzhou Institute for Advanced Study University of Chinese Academy of Sciences Hangzhou 310024 China; ^6^ Key Laboratory for Micro‐Nano Physics and Technology of Hunan Province College of Materials Science and Engineering Hunan University Changsha 410082 China; ^7^ Henan Key Laboratory of Diamond Optoelectronic Materials and Devices School of Physics and Engineering Zhengzhou University Zhengzhou 45000 China

**Keywords:** electric manipulations, optical manipulations, perovskite photodetectors

## Abstract

Photodetectors built from conventional bulk materials such as silicon, III–V or II–VI compound semiconductors are one of the most ubiquitous types of technology in use today. The past decade has witnessed a dramatic increase in interest in emerging photodetectors based on perovskite materials driven by the growing demands for uncooled, low‐cost, lightweight, and even flexible photodetection technology. Though perovskite has good electrical and optical properties, perovskite‐based photodetectors always suffer from nonideal quantum efficiency and high‐power consumption. Joint manipulation of electrons and photons in perovskite photodetectors is a promising strategy to improve detection efficiency. In this review, electrical and optical characteristics of typical types of perovskite photodetectors are first summarized. Electrical manipulations of electrons in perovskite photodetectors are discussed. Then, artificial photonic nanostructures for photon manipulations are detailed to improve light absorption efficiency. By reviewing the manipulation of electrons and photons in perovskite photodetectors, this review aims to provide strategies to achieve high‐performance photodetectors.

## Graphical abstract

This review summarizes the state‐of‐the‐art electrical and optical characteristics of typical perovskite photodetectors. The electrical manipulations with advanced device structures and optical manipulations with artificial photonic nanostructures are detailed to improve light absorption, photoelectric conversion, and carrier transmission performance in perovskite photodetectors. This review aims to provide strategies to achieve high‐performance photodetectors.

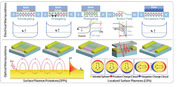

## 1 Introduction

Photodetectors can convert an optical (analog or digital) signal into an electrical signal, typically in the form of current or voltage. The fundamental mechanism in semiconductor photodetectors is the generation of electron–hole (e–h) pairs through the absorption of photons.^[^ ^1^, ^2^, ^3^, ^4^ ^]^ Photogenerated e–h pairs are then separated, collected, and transferred to external circuitry by an electric field, which is induced by an external voltage in a reverse‐biased junctions (as in p–n, p–i–n, and Schottky detectors) or in bulk (as in photoconductors).^[^ ^3^, ^5^, ^6^ ^]^ In some cases, photogenerated carriers can be amplified through external or built‐in gain processes.^[^ ^7^ ^]^

Ultraviolet–visible photodetectors based on silicon are currently facing further improvement demands for high quantum efficiency, miniaturization, and low power consumption. Meanwhile, infrared photodetectors based on III–V or II–VI compound semiconductors always have challenges of high preparation cost, low temperature operating requirements, and improvement of focal plane uniformity. As a result, there is an urgent need to develop new detection materials and manipulation mechanisms for high‐performance photodetectors. As direct bandgap semiconductors with high absorption coefficient and quantum efficiency, perovskite materials are attracting increasing attention not only in the field of solar cells but also in the photodetectors applications. Further, the facile and low‐cost preparation processes of perovskite materials provide an alternative material platforms for infrared photodetector development. However, to overcome the challenge of high dark current and low absorption efficiency, manipulations of electrons and photons on a local scale should be introduced in perovskite photodetectors. For example, electrical manipulations by gate electric field, built‐in electric field, and ferroelectric field can effectively suppress the dark current. The photogating electric field can lead to a high photoresponse by prolonging the carrier lifetime. The optical manipulations from surface plasmon polaritons (SPPs) and localized surface plasmons (LSPs) can enhance the light absorption due to electromagnetic oscillation caused by the interaction of free electrons and photons propagating at the interface of perovskite. Additionally, by coupling with other materials, the response spectrum of perovskite photodetectors could be expanded to the infrared range. In this review, we will discuss all of the above‐mentioned manipulations of electrons and photons on a local scale.

The chemical formula of perovskite materials is ABX_3_, where A is the monovalent cation such as Cs^+^, Rb^+^, CH_3_NH_3_^+^ (i.e., MA^+^) or HC(NH_2_)_2_^+^ (i.e., FA^+^), B is the bivalent metal cation (e.g., Pb^2+^, Sn^2+^, Bi^2+^ or Ge^2+^), and X is the halide anion (Cl^−^, Br^−^, and I^−^ or their mixtures).^[^ ^8^, ^9^ ^]^ Typical perovskite materials have an equiaxed crystal structure, which can be regarded as a grid frame composed of octahedrons in 3D space. To quantitatively describe the stability of perovskite materials, the tolerance factor *t* was proposed for the first time in 1926 to evaluate the relationship between structural stability and ion size, which can be expressed as *t* = (*R*_A_ + *R*_X_)/$\sqrt{2}$(*R*_B_ + *R*_X_), where *R*_A_, *R*_B_, and *R*_X_ are the corresponding ionic radius.^[^ ^10^ ^]^ Normally, *t* of perovskite materials with a stable structure is in the range of 0.78 to 1.05. For organic–inorganic hybrid perovskites, *t* is much different than 1 due to the large value of the ionic radius *R*_A_. As a result, perovskites will extend horizontally with the common vertex of divalent cations B and form a 2D structure.^[^ ^11^ ^]^ In the meantime, the organic linker layer of the 2D perovskite reduces the dielectric constant, which will result in a decrease of the induced charges produced in the perovskite.^[^ ^12^ ^]^ The weakened shielding effect with the decreasing of induced charges causes the Coulomb force between charged particles to increase and results in the enhancement of the binding energy between electron–hole pairs. Therefore, excitons instead of electron–hole pairs will be generated under illumination. The strong exciton binding energy can prevent the separation and collection processes of electron–hole pairs and result in poor photoresponsivity. Therefore, by introducing strong built‐in electric fields in the perovskite can break the exciton binding energy improving the photodetection performance.

Compared to 2D layered materials, perovskite materials are rarely studied for photodetection applications despite of their unique advantages. First, perovskite materials have a direct optical bandgap, which is independent of the material thickness leading to a high absorption coefficient and quantum efficiency. Second, the lower part of conduction band derived from the degenerated p bands with a lower dispersion than that of the delocalized s orbitals results in a higher density of states in the conduction band. Therefore, the efficiency of perovskites is much higher than that of other materials.^[^ ^13^ ^]^ On the other hand, with the quasi‐quantum well structure of inorganic and organic components, 2D perovskites can be produced as fullerene‐like crystals by mechanical exfoliation.^[^ ^14^, ^15^ ^]^ It allows integrating with other 2D materials by van der Waals force beyond the crystal lattice mismatching.^[^ ^16^, ^17^, ^18^, ^19^, ^20^ ^]^ This enables electrical and optical manipulations of electrons and photons in perovskite photodetectors.^[^ ^21^ ^]^ Moreover, an orthogonal processing and patterning method has been demonstrated to fabricate perovskite devices without compromising their electronic and optical characteristics. It overcomes the disadvantages that the 2D perovskites are easy to be damaged by the organic solvents in standard lithographic processes.^[^ ^22^ ^]^ Though several review papers on perovskite photodetectors are published recently, they focused on the material morphologies, device structures, performance, and function of the perovskite photodetectors. However, a few reviews focused on the fundamental mechanisms to improve device performance from the perspective of manipulations of electrons and photons on a local scale. Several of these effects are artificially introduced from structural design, while others are induced by the intrinsic characteristics of the perovskite materials. The strategies of forming these local fields will be summarized in this paper according to the evolution of perovskite photodetectors in recent years.^[^ ^23^, ^24^, ^25^ ^]^

**Figure 1:**
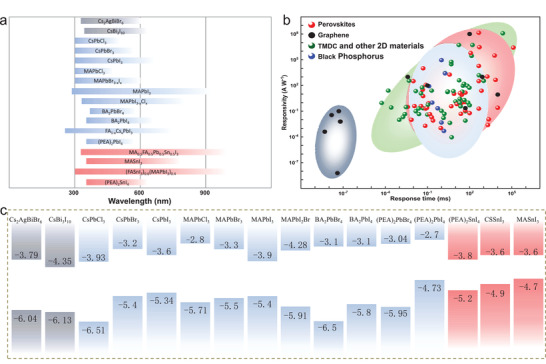
a) Detection ranges for different perovskite photodetectors. b) Comparison of the performances of photodetectors based on perovskite and low‐dimensional materials. c) Band structures for some common perovskite materials.

**Figure** **1** summarizes the band structures and detection wavelength ranges of different perovskites. The performance of perovskite photodetectors are compared with other low‐dimensional materials. The photoresponsivity and response time of perovskite photodetectors are between the transitional metal dichalcogenides (TMDs) and black phosphorus. Meanwhile, the detection wavelength range is challenged with the breakthrough for expanding to the infrared regimes. Early on, the perovskite photodetectors were mainly based on MAPbI_3_ structure. But the absorption region of pristine Pb‐based perovskites is mainly located in the range of ultraviolet and visible. However, the infrared band occupies nearly half of the energy in the solar energy spectrum. So, it is of great importance to further investigate the near‐infrared response for the perovskite photodetectors. On this basis, together with the toxicity of Pb, nontoxic Sn‐based perovskites that can extend the detection wavelength range to the near‐infrared regime is attracting more and more attention.^[^ ^26^, ^27^, ^28^ ^]^ However, stability is still the most challenging problem for perovskite‐based photodetectors.^[^ ^29^, ^30^, ^31^ ^]^ To address these problems, many research groups have tried Ge,^[^ ^32^ ^]^ Mn,^[^ ^33^ ^]^ Bi,^[^ ^34^, ^35^, ^36^ ^]^ and other elements as the metal cation for perovskites. Unfortunately, these perovskite materials still have some drawbacks, such as low photoresponsivity or high‐cost. Figure 1c summarizes band structures of perovskite materials. The location of the minimum energy of conduction band and the maximum energy of valence band is the basis of designing structure and optimizing device performance. The absolute band edge positions will determine the alignment of the bandgaps for perovskite heterojunctions and dictate charge transfer at the interface.

In this paper, the performance of perovskite photodetectors manipulated by the localized electric field and optical field is reviewed. The electric field manipulation includes traditional gate‐voltage, photogating effect, built‐in field, and ferroelectric field. The optical field manipulation makes full use of the plasmon enhancement, induced by the nanoparticles, gratings, antennas, photonic crystals, resonant cavities, and waveguides. In the following chapters, we will discuss how the electric field and optical field enhance the performance of perovskite photodetectors.

## 2 Fundamentals of Photodetectors

Photodetectors based on photoelectric effect include photoconductive, photovoltaic, photoelectromagnetic, Dember, and photon drag processes.^[^ ^37^, ^38^, ^39^, ^40^, ^41^, ^42^ ^]^ Among these photoelectric effects, photoconductive and photovoltaic (p–n junction and Schottky barrier) detectors are most commonly studied structures. In photovoltaic device, the optically injected excess carriers of opposite charges drift in opposite directions by built‐in electric field. In some applications, photovoltaic detectors are preferred over photoconductors because of their relatively high impedance, lower power dissipation, and faster response from the large velocity of photogenerated carriers in the depletion region.^[^ ^43^ ^]^

However, it is difficult to characterize performance parameters of infrared photodetectors due to the experimental variables involved. A variety of environmental, electrical, and radiometric parameters must be taken into consideration and carefully controlled. The characteristics of photodetectors based on novel complex structures has been even more complicated and demanding because of the local‐field‐effect.^[^ ^44^ ^]^ Here, some universal photodetector configurations will be described to discuss the improvement for photodetectors based on perovskites.

### 2.1 Responsivity and Quantum Efficiency

Considering that light with power *P* completely irradiates the effective detection area of the detector and produces a net photocurrent of *I*_ph_ or a photogenerated voltage of *V*_ph_, the responsivity of the device is defined as below

(1) $$R_{i}=I_{\mathrm{ph}}/P\left(A/W\right)\;\mathrm{or}\;R_{v}=V_{\mathrm{ph}}/P\left(V/W\right)\;$$

where *R*_i_ is the current responsivity, and *R*_v_ is the voltage responsivity. If the incident light is monochromatic, *R*_i_ can be expressed as

(2) $$R_{i}=\;\mathrm{EQE}\frac{q\lambda }{hc}$$

where *h* is the Planck constant, and *c* is the light speed. EQE is the external quantum efficiency, which represents the ratio of the number of excited electron–hole pairs to the number of incident photons. Considering the reflection and transmission of light, the internal quantum efficiency is IQE = EQE/*η*, where *η* is the absorption coefficient of light.

If the light source is broad‐spectrum, such as a black body, then the average responsivity of the band is

(3) $$R_{i}=\frac{\int_{\lambda_{1}}^{\lambda_{2}}R_{\lambda }\left(\lambda \right)\phi \left(\lambda \right)dy}{\int_{\lambda_{1}}^{\lambda_{2}}\phi \left(\lambda \right)dy}\;$$

where *ϕ*(*λ*) is the wavelength distribution of the irradiation power for incident light. *R_*λ*_* is the wavelength distribution of responsivity. *λ*_1_ and *λ*_2_ are the starting and cutoff wavelengths of the light source, respectively. The corresponding distribution of EQE could be deduced from *R_*λ*_*.^[^ ^45^ ^]^

### 2.2 Response Time and Bandwidth

The response time reflects the speed and application range of the detector. The responsivity of the modulation frequency *f* follows

(4) $$R\left(f\right)=\frac{R\left(0\right)}{\sqrt{1+4\pi^{2}f^{2}\tau^{2}}}\;$$

where *R*(0) is the responsivity of zero modulation frequency, and *τ* is the response time of the detector. The responsivity is almost independent of the frequency when the modulation frequency *f* is far less than 1/2*πτ*. As *f* becomes close to or larger than 1/2*πτ*, the responsivity of photodetectors decreases with the increase of modulation frequency *f* for incident light. The bandwidth of the detector is defined as the optical modulation frequency *f*_c_ with the response attenuation of 3 dB.^[^ ^7^ ^]^ The response time is defined as *τ* = 1/2*πf*_c_. Another common way to define the response time is by determining the time of photocurrent rising from 10% to 90% (rise time) or decreasing from 90% to 10% (fall time) with square‐wave modulated incident light. In actual experiments, these two methods had no significant difference from each other.

The response time of photoconductive detectors generally depends on the lifetime of photocarriers, and the response time of photovoltaic detectors mainly depends on the drift process of minority carriers in the junction region. Photodetectors based on perovskites have a longer response time than that of other low‐dimensional materials because the defect concentration and ion shielding effect result in a relatively long carrier lifetime. Recently, some perovskite photodetectors an ultrafast response of nanoseconds with the testing approach being transient photocurrent (TPC). The response time of TPC (also called decay time) is defined as the time for the photocurrent to decrease from the peak to ≈1/*e* after a single exponential fit for the TPC curve. But, the modulation laser for the TPC curve is a pulse light source and it is difficult to confirm the steady‐state response of saturation photocurrent. Consequently, the experimental response time obtained by the TPC approach could be possibly faster than those obtained by the standard square wave test method.^[^ ^46^ ^]^ If we strictly follow the definition of the response time for the photodetectors, a square wave test method is more appropriate.

### 2.3 Noise and Specific Detectivity

When the photodetector is functioning, the output current and voltage signals are fluctuating randomly with time. The random fluctuation part of the photodetector output is called “noise,” which is independent of the incident radiation statistics. The responsivity and quantum efficiency represent the signal intensity from the photodetector. The device detectivity is the comprehensive sum of the signal intensity and noise. It represents the sensitivity of the device. In this section, the thermal noise, shot noise, 1/*f* noise, and compound noise are introduced.

#### 2.3.1 Thermal Noise

The thermal noise is also known as Jones noise, which is the result of the random thermal motions of the charge carriers. Considering a cross‐section of the resistance, macroscopic statistics show that the carrier motion is isotropic, but microscopic statistics reveal the fluctuations of the carriers. The level of thermal noise is related to the absolute temperature *T*, even if the detector is not working under a bias voltage. The thermal noise voltage is^[^ ^47^ ^]^

(5) $$v_{j}={\left(4k_{B}TR\Delta f\right)}^{1/2}\;$$

where *R* is the detector resistance, and Δ*f* is the electronic bandwidth of noise measurement, therefore the thermal noise current can be expressed as

(6) $$\bar{i_{j}^{2}}=\frac{4k_{B}T\Delta f}{R}\;$$

The frequency limit of the thermal noise is ≈10^12^ Hz, which is much higher than that of the electronic acoustic bandwidth.^[^ ^48^ ^]^ As a result, the thermal noise is generally considered as white noise, which is unrelated to the electronic bandwidth.

#### 2.3.2 Shot Noise

The random fluctuation of current or voltage signal caused by free electrons and holes passing through the barrier or reaching the destination discontinuously is called shot noise. The shot noise increases with the current and obeys Poisson distribution

(7) $$\bar{i_{j}^{2}}=2qI\Delta f$$

where *I* is the average current.

#### 2.3.3 Generation‐Recombination (G‐R) Noise

In photoconductive devices, the lifetime of photogenerated carriers are a random variable of the average value $\bar{\tau }$. The noise caused by this mechanism is called G‐R noise, and the noise current is^[^ ^49^ ^]^

(8) $$\bar{i_{\mathrm{gr}}^{2}}=\frac{4qI_{\mathrm{ph}}g\Delta f}{1+{\left(2\pi f\tau \right)}^{2}}\;$$

where *I*_ph_ is the average net photocurrent, *g* represents the photoconductive gain, and *f* is the noise spectrum frequency. Obviously, G‐R noise is not white noise, which is dependent on frequency. When *f* ≪ 1/*τ*, the noise current is abbreviated as^[^ ^50^ ^]^

(9) $$\bar{i_{\mathrm{gr}}^{2}}=\;4qI_{\mathrm{ph}}g\Delta f$$

#### 2.3.4 1/*f* Noise

The 1/*f* noise is a type of low‐frequency noise, which is quite possibly originating from the fluctuation of conductivity induced by the electron concentration change. The internal mechanism for the low frequency dependence of noise is not completely clear. Some of the publications summed up that the 1/*f* noise is largely induced by surface‐related noise, such as generation‐recombination, tunneling, diffusion, and shunt noise.^[^ ^51^, ^52^, ^53^ ^]^ Here, we present the empirical formula of the noise spectrum density, which is inversely proportional to the frequency^[^ ^54^ ^]^

(10) $$\bar{i_{1/f}^{2}}=\;k\frac{I^{b}}{f^{a}}\Delta f$$

where *k* is a proportional constant, and *a* and *b* are related parameters.

In addition to the basic noise sources introduced above, the dark current, temperature noise, photon noise, and other sources are also common noise factors. In general, the focal plane circuits need to integrate the photocurrent of the device through capacitance coupling, so the dark current is an important contributor to overall system noise. The ambient temperature is not a constant value, which will cause fluctuations in the internal noise of the device. Additionally, because the photons are quantized, the fluctuation in the number of incident photons will lead to the corresponding shot noise.

The noise of the detector should be the sum of all these noise sources.

The noise equivalent power (NEP) is defined as the incident light power when the signal‐to‐noise ratio is equal to 1 in a 1 Hz bandwidth. In general, the response rate can be used to express the noise equivalent power

(11) $$\mathrm{NEP}\;=\frac{i_{n}}{R_{i}}\;=\frac{v_{n}}{R_{v}}\;$$

where *i*_n_ and *v*_n_ are the total noise signal of different components.

#### 2.3.5 Specific Detectivity

The specific detectivity can be defined as

(12) $$D^{*}=\frac{\left(A\Delta f\right)}{\mathrm{NEP}}=\frac{\left(A\Delta f\right)R_{i}}{i_{n}}=\frac{\left(A\Delta f\right)R_{v}}{v_{n}}$$

where *A* is the effective detection area of the detector, and Δ*f* is the bandwidth of the test circuit. The detectivity takes the photosensitive element area into account, which is a comprehensive index to compare the sensitivity of two detectors. With different working temperatures and bias voltages, the influence of different mechanical components on noise cannot be generalized. However, some implementations of low‐dimensional photodetectors use the dark current at zero bias as the noise current to estimate D*, which is improper. As a result, a variety of experimental results were reported, which exceeded the theoretical limit of D*.

Several important performance parameters of photodetectors are introduced, including response rate, response time, noise, and detectivity. These provide a reference for evaluating the performance of photodetectors based on perovskites. Besides, the working temperature, cut‐off wavelength, and dynamic range of the photodetectors are also very important.^[^ ^55^ ^]^

## 3 Electric Field Manipulations

**Figure 2:**
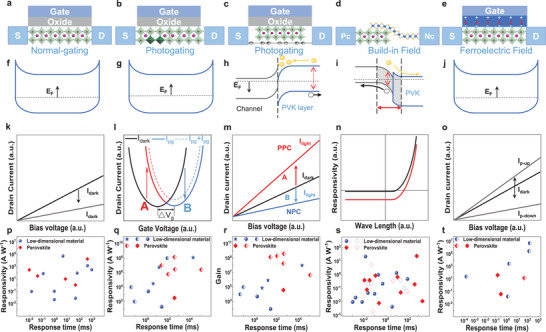
Perovskite photodetectors of different electric manipulations. a–e) Device structures of perovskite photodetectors with gate voltage field, photogating effect, built in electric field, and ferroelectric field manipulations. f–j) Schematic diagram of band structure for perovskite photodetectors with gate voltage field, photogating effect, built in electric field, and ferroelectric field manipulations. k–o) Typical characteristic curves for perovskite photodetectors with gate voltage field, photogating effect, built in electric field, and ferroelectric field manipulations. p–t) Performance for perovskite photodetectors compared with traditional low‐dimensional photodetectors manipulated by gate voltage field, photogating effect, built in electric field, and ferroelectric field. The red diamonds refer to perovskite photodetectors, the blue circles refer to 2D photodetectors, and the blue stars refer to nanowire photodetectors. The solid points represent manipulation mechanism within single materials, the half‐solid points represent manipulation mechanism of hybrid structures, and the hollow points represent manipulation mechanism of Schottky junction.

For photodetectors with a well‐defined material consistency, maximizing the performance of materials is the primary task of device design and preparation. Electric field manipulation is an important link between device design and optimization. In this section, the mechanism and performance of perovskite photodetectors manipulated by the localized electric field are reviewed. The localized electric field includes gate voltage electric field, photogating electric field, built‐in field, and ferroelectric field (**Figure** **2**).

### 3.1 Manipulation of Gate Voltage Field

The typical structure for manipulation of the gate voltage field is a three‐terminal transistor, which consists of a source, drain, and gate electrode. The schematic and energy band diagrams of gate voltage manipulation are shown in Figure 2a,f. Under gate field modulation, the Fermi level of perovskites can be increased or decreased. As a result, the carrier concentration in dark conditions can be suppressed (Figure 2k). Therefore, an appropriate gate‐voltage‐field can effectively improve the responsivity by reducing the dark current.

For perovskites, ion migration will screen the gate electric field, making the manipulation of carriers more difficult than in other materials.^[^ ^56^, ^57^ ^]^ In order to realize gate voltage modulation, several approaches have been adopted to reduce the ion migration, such as reducing the working temperature,^[^ ^58^, ^59^ ^]^ introducing less‐volatile inorganic cations into perovskites,^[^ ^60^, ^61^ ^]^ reducing the material thickness,^[^ ^62^ ^]^ and utilizing external light source acts as the fourth terminal electrode.^[^ ^63^ ^]^

In 2015, Li et al. demonstrated a phototransistor based on MAPbI_3_ perovskites film materials.^[^ ^63^ ^]^ Through precise control of the perovskites thickness, high gate‐field manipulated photoresponsivity of 320 A W^−1^ was achieved under −40 V gate voltage. It is noteworthy that the thickness of perovskites is very important here. If the thickness is too thin, the absorption coefficient of the photodetector will be limited. If the thickness is too thick, the electrostatic shielding effect will be conspicuous. In 2017, a phototransistor based on (PEA)_2_SnI_4_ was reported with the improved responsivity of 1.9 × 10^4^ A W^−1^, which is the highest responsivity of this material achieved so far.^[^ ^62^ ^]^

Figure 2p presents the performance for most perovskite photodetectors compared with low‐dimensional photodetectors manipulated by the gate voltage field (the solid circle represents low‐dimensional photodetectors, and the solid diamond represents the perovskite photodetectors).^[^ ^62^, ^63^, ^64^, ^65^, ^66^, ^67^, ^68^, ^69^, ^70^, ^71^, ^72^, ^73^, ^74^ ^]^ Although perovskite photodetectors have not been studied for a long time, the responsivities with gate‐voltage‐field manipulation have been at par with some excellent low‐dimensional photodetectors.

### 3.2 Manipulation of the Photogating Field

The photogating effect is quite common in traditional photoconductive devices. However, it has little impact on conventional bulk materials, because artificial traps can hardly affect the overall carrier distribution in the bulk semiconducting channel. As the device channel approaches micro or nanoscale, these trap‐states are strong enough to perform the “fulcrum effect.” The underlying mechanism for photogating is that the trapped electrons/holes act as a local gate and modulate the channel carrier concentration. This is the main difference from the common photoconductive gain, where the photoconductivity is directly generated by the separation of photogenerated carriers in the channel.^[^ ^75^ ^]^ Normally, the photocurrent of low‐dimensional photodetectors manipulated by photogating is a combination of photogating and photoconductive gain. But in the thin film materials, the photogating mechanism is easily masked by the photoconductive gain effect.

The net photocurrent of the photogating effect can be expressed as

(13) $$I_{\mathrm{ph}}=\frac{\partial I_{d}}{\partial V_{g}}\Delta V_{g}=g_{m}\Delta V_{g}$$

where Δ*V*_g_ is the photogenerated potential, and *g*_m_ is the transconductance of the phototransistor. The responsivity of photodetectors dominated by photogating is much higher than 10 A W^−1^.^[^ ^76^, ^77^, ^78^, ^79^ ^]^ Combined with an excellent absorption coefficient of perovskite materials, the responsivity of photodetectors can reach 10^9^ A W^−1^.^[^ ^80^ ^]^ However, the high responsivity comes at the expense of device bandwidth, which is less than 1 MHz.

Figure 2b,c presents the photogating structure based on a single perovskite and hybrid structure. The main differences between these two structures are the separation or merging of the channel layer and gating area.^[^ ^75^ ^]^ However, there is no significant difference in the manipulation mechanism of these two structures. Under illumination, one type of carrier will be trapped in localized states, either in the channel layer or adjacent to the channel layer.^[^ ^81^, ^82^ ^]^ The electric field effect of the trapped carriers will then shift the Fermi level and gate the conduction of the channel (Figure 2g,h).

Figure 2l indicates the shifts of the transfer characteristic curve for transistors under illumination.^[^ ^83^ ^]^ Here, two mechanisms of positive photoconductivity (PPC) and negative photoconductivity (NPC) are present. The photocurrent at the voltage of point A is greater than the dark current and presents a PPC. The NPC is at the voltage of point B, whose photocurrent is less than the dark current. The *I*–*V* characteristic curves for both PPC and NPC at points A and B are shown in Figure 2m.

**Figure 3:**
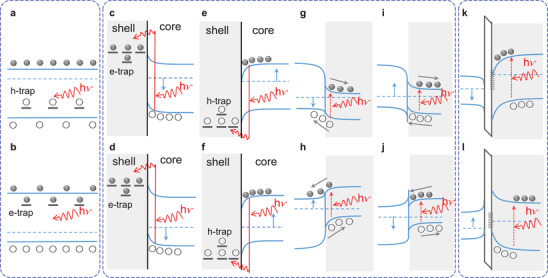
Photogating mechanism in three types of structures. The shaded part represents the gating layer. a,b) Photogating mechanism occurring in a single material with hole‐ or electron‐trap center. c–f) Photogating mechanism based on core–shell structure with a gating layer of insulator. The electron‐traps in the gating layer decrease the Fermi level and result in a negative response for n‐type semiconductor or a positive response for a p‐type semiconductor. The hole‐traps in the gating layer increase the Fermi level and result in a positive response for n‐type semiconductor or a negative response for a p‐type semiconductor. g,h) Photogating mechanism based on type II heterojunction. The photoinduced carriers transfer into the channel and change the response of the perovskite. i,j) Photogating mechanism based on type I heterojunction. The trapped carriers at the interface could result in a positive or negative response of the photodetector. k,l) Photogating mechanism in hybrid structure of three materials. Here, the photoinduced carriers are blocked by the infinite high barrier and trapped at the interface. The trap‐state will act as a gating role to manipulate the conduction characteristic of the channel.

Up to now, the photogating mechanism has been found in three types of structures, which are shown in **Figure** **3**. Figure 3a,b presents the photogating effect occurring in a single material. Figure 3c–j is the band characteristics of photogating based on a hybrid structure of two materials. Figure 3k,l displays the photogating mechanism in a hybrid structure of three materials.

The photogating effect in a single material is a typical photoconductive‐type of photogating (Figure 3a,b). Here, one type of the carriers is trapped by the defects in the shallow energy level, which results in a prolonged minority carrier lifetime, and provides the photogating behavior. The gain in this mechanism is positively related to the majority carrier lifetime. It can be expressed as *G* = *τ*/*τ*_*t*_, where *τ* is the minority carrier lifetime, and *τ*_*t*_ is the transit time for the carrier. This type of photogating has a faster response time than the photovoltaic type, but the gain is relatively small.

In the second type, the photogating effect in the channel can be induced by the gating layer of an insulator or semiconductor. If the gating layer is an insulator, the electron‐traps in the gating layer will result in a decrease of the Fermi level in the channel layer (Figure 3c,d). Then, a negative/positive response will be induced for a n/p‐type channel (Figure 3c,d). Figure 3e,f shows the gating layer with hole‐traps. If the grating and channel regions are both semiconductors, the heterojunction type of the two semiconductors will determine the response. For the type II heterojunction, one type of the photoinduced carriers excited in the gating layer will transfer into the channel and manipulate the carrier concentration of the channel. Figure 3g,h presents the positive response for the type II heterojunction with p–n and n–p junction. For the type I heterojunction, the photoinduced carriers excited in the gating layer cannot be transferred into the channel because the carriers will be blocked and trapped by the band offset. The accumulated carriers in the potential well will provide a gating effect to the carriers in the channel. Figure 3g,h presents the negative response for the type I heterojunction with p–n and n–p junction.

In the third type of photogating structure, there is an insulating layer between the channel and the photon absorption layer. As a result, one type of the photoinduced carriers will be blocked and trapped by the infinite high barrier. The locally trapped electrons will decrease the Fermi level of the channel and provide a positive response for the p‐type channel or a negative response for the n‐type channel. The local trapped holes can provide an opposite situation for the channel layer.

So far, these three types of photogating manipulation have been used in novel photodetectors and the appropriate design of photogating can be applied to improve the device sensitivity. In 2014, the photogating mechanism was first introduced in the perovskites–graphene hybrid system by Lee et al.^[^ ^84^ ^]^ Figure 2q,r shows the comparison of perovskite photodetectors with low‐dimensional photodetectors manipulated by the photogating effects.^[^ ^85^, ^86^, ^87^, ^88^, ^89^, ^90^, ^91^, ^92^, ^93^, ^94^, ^95^, ^96^, ^97^, ^98^ ^]^ The solid symbols represent the photogating effect from internal defects, and the half‐solid symbols represent photogating in a hybrid structure. Perovskite photodetectors with photogating mechanism demonstrate advantages in terms of responsivity and gain and disadvantages in terms of response time.

### 3.3 Manipulation of the Built‐In Electric Field

Heterojunctions have become a classic structure in optoelectronic devices since H. Kroemer and Z. I. Alferov were granted Nobel Prize in Physics for the heterojunction transistor and laser diode. The built‐in electric field in the heterojunction can accelerate the separation efficiency of photoexcited electron–hole pairs. The fast separation process of photogenerated electron–hole pairs in the space charge region results in an ultrafast response speed. In addition to response time, the low dark current is another advantage for the heterojunction photodetectors. First, the built‐in electric field can suppress majority carrier diffusion and avoid the trap‐assisted‐tunneling, band to band tuning, and other generation‐recombination noise from applied large bias voltage. On the other hand, the dark current of the photodetector is further suppressed by the large resistance in the space charge region forming a built‐in electric field. As can be seen, the manipulation of a built‐in electric field has the natural advantages of ultralow dark current allowing superior detectivity in heterojunction photodetectors compared with other gain devices.

Many of the recent studies on perovskite heterojunction photodetectors show advantages in terms of response time and ultralow dark current with reduced charge recombination.^[^ ^99^, ^100^ ^]^ Additionally, the response spectra of perovskite‐based photodetectors could be extended from the ultraviolet region to the near‐infrared range by integrating with other narrow‐bandgap materials. For example, Dou et al. constructed built‐in electric field in perovskite heterojunction photodetectors showing high detectivity of up to 10^14^ Jones, a linear dynamic range over 100 dB, and a fast response with 3 dB bandwidth up to 3 MHz at room temperature.^[^ ^101^ ^]^ A broadband response spectra up to 1 µm with an external quantum efficiency of 54% at 850 nm.^[^ ^46^ ^]^

For heterojunction perovskite photodetectors, the interface states is unavoidable and cannot be ignored. For example, Shewmon et al. adopted a graded layer at the interface between MAPbI_3_ and MAPbBr_3_, which resulted in a responsivity of 0.03 A W^−1^ at 500 nm.^[^ ^102^ ^]^ Wu and co‐workers provided a perovskite/graphene heterostructure with chemical vapor deposition selective growth of MAPbBr_3_ platelets on the graphene which presents a novel platform for investigating the interfacial coupling of perovskite heterojunctions.^[^ ^103^ ^]^ These studies demonstrate that suitable interface materials and construction methods are effective approaches to improve the performance of perovskite photodetectors.

Figure 2s indicates the performance for most perovskite photodetectors compared with low‐dimensional photodetectors manipulated by the built‐in field. (The solid circles represent low‐dimensional photodetectors, the solid diamonds represent the perovskite photodetectors, and the hollow points represent the photodetectors of the built‐in electric field from Schottky junction.)^[^ ^20^, ^46^, ^104^, ^105^, ^106^, ^107^, ^108^, ^109^, ^110^, ^111^, ^112^, ^113^, ^114^, ^115^, ^116^, ^117^, ^118^, ^119^, ^120^, ^121^, ^122^, ^123^, ^124^, ^125^, ^126^, ^127^, ^128^, ^129^, ^130^ ^]^ The response time of photodetectors with a built‐in electric field reveals distinct advantages compared with other manipulation mechanisms and compensates for the response‐speed deficiency of perovskite photodetectors.

### 3.4 Manipulation of a Ferroelectric Field

Ferroelectricity, as one of the important properties of perovskites, cannot be omitted in the manipulation of the electric field. This fascinating feature will open extensive perspectives for the assembly of high‐performance optoelectronic devices. Previously, the ferroelectric effect has been employed to depress the dark current of 2D semiconductors with an ultrahigh local electrostatic field (≈10^9^ V m^−1^ within several nanometer scales) from the stable remnant polarization.^[^ ^131^ ^]^ However, realized designs of hybrid and pure perovskite ferroelectric photodetectors have rarely been reported (Figure 2t).^[^ ^85^, ^131^, ^132^, ^133^, ^134^, ^135^, ^136^, ^137^ ^]^

Chirality is another important intrinsic characteristic for some types of perovskite. Based on chiral recognition, direct detection of circular polarized light has been realized for perovskite photodetectors without optical polarizers. The polarization discrimination ratio could be as high as 25.4.^[^ ^137^ ^]^ These electric manipulations based on the intrinsic characteristics of the perovskites could greatly decrease the complexity of a perovskite detection system.

In conclusion, electric manipulation of photogating reveals significant advantages for responsivity among all the electric field manipulations. However, response time is still a problem in this mechanism. The manipulation of built‐in electric fields could provide an excellent response time, but with poor responsivity. The trade‐off between responsivity and response time demands scrutiny for appropriate applications. On the other hand, designing an ingenious structure to mitigate the trade‐offs can provide another opportunity for the development of perovskite photodetectors. This breakthrough has already been realized by Guo et al. with a light‐driven junction of traditional 2D material.^[^ ^138^ ^]^ Additionally, the spontaneous polarization of perovskite, which can provide natural polarization characteristics and dark current suppression effect, has not been completely utilized. All in all, the electric manipulation of perovskite photodetectors still leaves us a large space for exploration and performance‐improvement.

## 4 Optical Manipulations

Optical manipulation is another dimension that can be used to improve the photoresponse and suppress the dark current. Moreover, along with devices becoming more and more miniaturized and perovskite material gradually evolving to micro–nanostructures, the limited thickness of the absorption layer leads to the requirement for an increase in optical manipulations. In this section, the mechanism and structures of optical manipulations for perovskite photodetectors are discussed, including SPPs, LSPs, photonic crystals, resonant cavities, and waveguides.

### 4.1 Manipulation Based on SPPs and LSPs

SPP is electromagnetic oscillation caused by the interaction of free electrons and photons propagating at the interface between the microstructure (conductor) and absorbing layer (dielectric). But the electromagnetic oscillation of the LSP is localized without propagation in the sealed surface of the microstructures.

**Figure 4:**
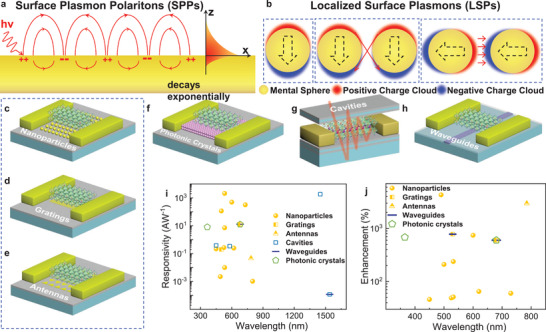
Perovskite photodetectors with different optical manipulations. a) SPP caused by the interaction of free electrons and photons propagating at the interface between the metal structure and perovskite. b) Localized electromagnetic oscillation of the LSP in the seal surface of the microstructures without propagation. c–e) Typical optical manipulation structures of nanoparticles, gratings, and antennas for perovskite photodetectors based on SPP and LSP. f) Optical manipulation structure of photonic crystal. g) Optical manipulation structure of photonic crystal. h) Typical optical manipulation structure of waveguide. i,j) Responsivity and enhancement of the published perovskite photodetectors with different optical enhancement. Here, the yellow point refers to performance of perovskite photodetectors based on the nanoparticle, the yellow square refers to the manipulation of grating, and the yellow triangle refers to the antenna. The hollow pentagon represents the perovskite photodetectors with photonic crystals. The hollow square represents the manipulation of resonant cavities. The stick represents waveguides. The overlapped points in the graph indicate the multiple optical manipulation mechanisms within one perovskite photodetector.

As shown in **Figure** **4**a, the electromagnetic wave of SPPs arises via the coupling of the electromagnetic fields to oscillations of the conductor's electron plasma. It propagates along the interface and decays exponentially in the direction of the vertical interface. Recently, low‐dimensional perovskites with natural a nanoscale organic passivation layer avoid the manual passivation structure, which will keep the absorption layer away from the artificial nanostructure and limit the light absorption enhancement. The light field enhanced structure based on SPPs includes antennas and gratings.

Figure 4b presents the second fundamental excitation of plasmonics, LSPs. The resonance of LSPs arises from the imbalance of the restoring force on the driven electrons and nanoparticle‐curved‐surface, which will lead to field amplification both inside and in the near‐field zone outside the nanoparticle. Here, if the arrangement of the nanoparticles is sparse, the interparticle coupling could be neglected. But, if the nanoparticles are arranged closely, the interparticle coupling will lead to shifts of the plasmon resonance. The shift depends on the polarization direction of the exciting light. For excitation of transverse modes, the increased restoring force acting on the oscillating electron of nanoparticles will lead to a blue‐shift. While the decreased restoring force for excitation of longitudinal modes will lead to a redshift. Based on the LSPs, different nanoparticles and antennas are designed to enhance the absorption of the photodetectors.

So far, most of the optical manipulation for perovskite photodetectors is based on nanoparticles (Figure 4c). For example, the first perovskite photodetector demonstrated with plasmonic enhancement by Zheng and co‐workers in 2016 is constructed with Au nanocrystals. It was evidenced by a 238% plasmonic enhancement factor and 10^6^ on/off ratio.^[^ ^139^ ^]^ Following this work, enhanced structures of Au nanoparticles, Ag nanoparticles, and Au nanorods for perovskite photodetectors were published respectively in 2016, 2017, and 2019.^[^ ^140^, ^141^, ^142^, ^143^, ^144^, ^145^ ^]^ Especially, in 2017, Kim and co‐workers compared the Ag nanoparticle‐, Au nanoparticle‐, and Au nanoparticle‐mediated devices, which revealed different enhancements of responsivity.^[^ ^141^ ^]^ However, the controlling variables of size, shape, metallic material, and incident light for the nanoparticles in this work were short of a rigorous design. J. Wu's work in 2020 made up for the deficiency of this work. The latter work conducted a systematic study on the enhanced structure of Ag, Cu, and Al nanoparticle/AAO hybrid plasmonic nanostructures. The work demonstrates the strong geometric dependence of silver nanoparticles/AAO hybrid plasma nanostructures on performance.^[^ ^146^ ^]^ The Al nanoparticle/AAO hybrid plasma nanostructure achieves the best enhancement of 4300%.^[^ ^147^ ^]^ Except for the responsivity enhancement, Fang and co‐workers broadened the absorption range to the near‐infrared range by manipulating the size of the nanosquare arrays.^[^ ^147^ ^]^

Aside from the nanoparticles, the LSP antenna structure has also been realized by Guo and co‐workers in 2020 (Figure 4d).^[^ ^148^ ^]^ An enhanced electric field from the plasmonic integration in this work increases light absorption through the LSP coupling of Au bowtie antenna arrays and the incident optical radiation. The photoresponsivity under 785 nm laser illumination is demonstrated with an enhancement factor of 2962%. This work is one of the few antennae enhanced perovskite photodetectors so far.

The grating couple is another common SPP structure for propagating the 2D electromagnetic waves at the interface between the optical structures and absorbing materials (Figure 4e). The grating of grooves or holes with lattice constant *α* could also overcome the mismatch in wave vectors between the in‐plane momentum of impinging photons and the propagation constant *β*. Li and co‐workers imprinted a DVD‐R disc on the CsPbBr_3_ layer and constructed an appropriate grating structure of a perovskite photodetector, which can increase the light path length and form convergence in the perovskite.^[^ ^149^ ^]^ A high responsivity of 0.25 A W^−1^ is acquired in this work. However, the high‐performance in this work is mainly attributed not only to the optical manipulation of the grating but also to the coupling of the conjugated‐polymer. The enhancement of the grating structure for perovskite photodetectors has not been mentioned in this work.

### 4.2 Manipulation of Photonic Crystals

Besides the structures based on SPPs and LSPs, the photonic crystal is another major structure that can manipulate the distribution of light (Figure 4f). The photonic crystal is a periodic nanostructure of two interpenetrating domains with different dielectric constants and refractive indices. The periodicity for the periodic domain is based on the wavelength of light. By controlling the periodic structure, the spatial distribution of an electromagnetic field can be manipulated. It results in a local field enhancement in one of the dielectrics in the periodic domain.

Recently, only a few studies of perovskite photodetectors concentrate on the photonic crystals induced enhancement mechanism. The representative publication in recent years is the ultraviolet photodetector of blue perovskite enhanced by the polymethyl methacrylate (PMMA) opal photonic crystals effect and Ag plasmon effect.^[^ ^150^ ^]^ Matching the plasmon peak of Ag film and photonic stop band of PMMA opal photonic crystals with the emission peak of blue CsPbCl_3_, enhanced the photocurrent by 682%.

### 4.3 Manipulation of Resonant Cavities

The enhancement for resonant‐cavity‐manipulation is achieved by inserting the perovskite material into a resonant cavity composed of top and bottom mirrors (Figure 4g). At the resonance condition, the incident light interferes constructively with the reflected component of the bottom mirror. After multiple reflections in the cavity, the resonant radiation results in the enhancement of the internal optical field. The low‐dimensional photodetectors of organo–inorganic hybrid perovskite especially benefit from the enhanced resonant field, in which every resonant photon equivalent traverses the cavity multiple times. The absorption coefficient of low‐dimensional materials is usually limited as the size of at least one dimension in low‐dimensional materials is reduced below the detection wavelength. With the enhanced resonant field of cavities, the low‐dimensional perovskite serves many times in generating photocarriers thus compensating for the low absorption coefficient of low‐dimensional materials. Additionally, the wavelength selectivity, in which only resonant wavelengths are admitted, makes the resonant cavities attractive for monolithic integration of photodetectors with the filter.

Based on the advantage of the field amplification and wavelength selectivity for the resonant cavities, different structures and resulting performance of perovskite photodetectors have been recently published. In A. L. Pan's work in 2017, the perovskite serves as a waveguide cavity, which can efficiently confine and transport light.^[^ ^151^ ^]^ A significant responsivity and external quantum efficiency are present in this work. Additionally, with upconversion luminescence confined in the cavities and coupled into perovskite, the spectral response for the perovskite photodetector has been expanded to 1.54 µm for the first time. Fang and co‐workers presented an excellent responsivity of 1.84 × 10^3^ AW^−1^ for CsPbI_3_ nanotubes with the light‐trapping enhancement within a tube cavity in 2019.^[^ ^152^ ^]^ Except for the absorption and response enhancement, Chen and co‐workers fabricated a perovskite color light detector with a three‐resonant cavity structure that served as a white light filter of 400 to 750 nm.^[^ ^153^ ^]^

### 4.4 Manipulation of Waveguides

The waveguide is another mechanism of optical manipulation that offers the promise for the development of multiple sensors on one chip (Figure 4h). The trade‐off between confinement and propagation loss in the waveguide technology demands an advisable choice of geometry, depending on the length of the energy transfer path.

Recently, many research groups are dedicated to integrate optical waveguides into advanced materials. This manipulation mode with special application requirements has been gradually realized in perovskite photodetectors. Song and co‐workers confirmed the conversion of incident light from propagating waves to guiding waves in 2017.^[^ ^154^ ^]^ As a result, the absorptivity is significantly increased with the absorbing length changed from the device thickness to the waveguide length. Except for the waveguide mode, a grating coupler in this work also contributed to the performance of the perovskite photodetector. With the combination of waveguides and gratings manipulation, the photocurrent of this organometallic halide perovskite photodetector has been enhanced by 780%. Beyond the unique manipulation with two types of optical fields, Li and co‐workers fabricated a hierarchical light‐trapping architecture with grating diffraction, waveguide mode, and reflection of the photonic crystal stopband. This work was inspired by the elaborate architecture of butterfly *Papilio paris* in 2019.^[^ ^155^ ^]^ Here, the waveguide layer is sandwiched between the grating layer and the photonic crystal layer because effective refractive indexes for grating and photonic crystal layers with porous structures are lower than that of the grained perovskite layer in the middle. With the coupling effect of the light‐trapping antireflective ability from three types of optical manipulation, the photocurrent has been enhanced by more than 600%.

In conclusion, optical manipulation is an important dimension to improve the performance of perovskite photodetectors. Figure 4i,j presents the responsivity and enhancement of the published perovskite photodetectors incorporating optical enhancement. The nanoparticle is one of the most commonly used structures for perovskite photodetectors at present, which could be attributed to the simplicity and compatibility of the process. Among these perovskite photodetectors with nanoparticle enhancement, some of the research present very outstanding performance. In addition to nanoparticle enhancement, optical manipulation based on cavities provides for the possibility of super bandgap detection, which could compensate for the deficiency of infrared response in perovskite photodetectors. Besides, many researchers initiated a compound manipulation based on waveguides, photonic crystals, cavities, and so on. Such compound manipulation mechanisms will provide more possibilities for light field enhancement.

## 5 Conclusion and Perspective

In this review, we comprehensively discuss manipulations of electrons and photons in perovskite photodetectors. Among them, photogating, built‐in electric field, ferroelectric field, and gate field can precisely manipulate electrons that either improve responsivities or suppress dark current by changing the carrier transport of the perovskites. As a result, photodetectors manipulated by electric field on a local scale have the advantages of low power consumption, high response speed, low dark current, and high responsivity. The photogating field provides higher carrier lifetime which results in higher responsivities. The built‐in electric field can efficiently separate electron–hole pairs and to suppress diffusion of the majority carriers leading to a high response speed and low dark current. Finally, the ferroelectric field and gate voltage can suppress dark current to reduce the noise of devices. In addition to the electrical manipulations, optical manipulation is another important dimension for high‐performance photodetectors. The remarkable optical characteristics of tunable exciton binding energy and excellent nonlinear optical gain make perovskite an excellent candidate for optical manipulation. Here, the mechanism and structures of optical manipulations based on the nanoparticles, antenna effect, grating couple, photonic crystal resonant cavity, and waveguide have been summarized. All the manipulations can enhance the optical field in the perovskite and result in high quantum efficiency.

Perovskite photodetectors with artificial structures have demonstrated excellent control capability of the local field. In the future, the combination of photon and electron manipulations is bound to become a promising research area for high‐performance photodetectors. These methods can be applicable for most low‐dimensional materials.

## Conflict of Interest

The authors declare no conflict of interest.

## Author Contributions

F.W., X.Z., M.X., and H.W. contributed equally to this work. W.H. and L.L. conceived and supervised the project. F.W., M.X., H.W., and W.H. wrote the manuscript. All authors discussed the results and revised the manuscript.
